# Biosafety and Proteome Profiles of Different Heat Inactivation Methods for Mycobacterium tuberculosis

**DOI:** 10.1128/spectrum.00716-21

**Published:** 2021-12-22

**Authors:** Cheng-Hui Wang, Denise Utami Putri, Jau-Ching Lee, Chi-Chih Liao, Sung-tzu Tsao, Ai-Lin Hsiao, Jhao-Hui Wu, Xiao-Wei Chen, Chih-Hsin Lee, I-Lin Tsai

**Affiliations:** a Department of Laboratory Medicine, Wan Fang Hospital, Taipei Medical University, Taipei, Taiwan; b School of Medical Laboratory Science and Biotechnology, College of Medical Science and Technology, Taipei Medical University, Taipei, Taiwan; c Pulmonary Research Center, Wan Fang Hospital, Taipei Medical University, Taipei, Taiwan; d Division of Pulmonary Medicine, Department of Internal Medicine, Wan Fang Hospital, Taipei Medical University, Taipei, Taiwan; e Division of Pulmonary Medicine, Department of Internal Medicine, School of Medicine, College of Medicine, Taipei Medical University, Taipei, Taiwan; f Department of Biochemistry and Molecular Cell Biology, School of Medicine, College of Medicine, Taipei Medical University, Taipei, Taiwan; g Graduate Institute of Medical Sciences, College of Medicine, Taipei Medical University, Taipei, Taiwan; h Master Program in Clinical Genomics and Proteomics, School of Pharmacy, Taipei Medical University, Taipei, Taiwan; i International PhD Program for Cell Therapy and Regeneration Medicine, College of Medicine, Taipei Medical University, Taipei, Taiwan; j School of Pharmacy, Taipei Medical University, Taipei, Taiwan; University of Arizona/Banner Health

**Keywords:** *Mycobacterium tuberculosis*, heat inactivation, proteomics, biosafety, mass spectrometry

## Abstract

Studies involving the pathogenic organism Mycobacterium tuberculosis routinely require advanced biosafety laboratory facilities, which might not be readily available in rural areas where tuberculosis burdens are high. Attempts to adapt heat inactivation techniques have led to inconsistent conclusions, and the risk of protein denaturation due to extensive heating is impractical for subsequent mass spectrometry (MS)-based protein analyses. In this study, 240 specimens with one or two loops of M. tuberculosis strain H37Rv biomass and specific inactivated solutions were proportionally assigned to six heat inactivation methods in a thermal block at 80°C and 95°C for 20, 30, and 90 min. Twenty untreated specimens served as a positive control, and bacterial growth was followed up for 12 weeks. Our results showed that 90 min of heat inactivation was necessary for samples with two loops of biomass. Further protein extraction and a matrix-assisted laser desorption ionization–time of flight (MALDI-TOF) MS assay demonstrated adequate scores for bacterial identification (≥1.7), with the highest score achieved in the 80°C/90 min and 95°C/30 min treatment groups. A proteomics study also confidently identified 648 proteins with ∼93% to 96% consistent protein abundances following heating at 95°C for 20, 30, and 90 min. Heat inactivation at 95°C for 90 min yielded the most quantifiable proteins, and a functional analysis revealed proteins located in the ribosomal subunit. In summary, we proposed a heat inactivation method for the M. tuberculosis strain H37Rv and studied the preservation of protein components for subsequent bacterial identification and protein-related assays.

**IMPORTANCE** Inactivation of Mycobacterium tuberculosis is an important step to guarantee biosafety for subsequent M. tuberculosis identification and related research, notably in areas of endemicity with minimal resources. However, certain biomolecules might be denatured or hydrolyzed because of the harsh inactivation process, and a standardized protocol is yet to be determined. We evaluated distinct heating conditions to report the inactivation efficiency and performed downstream mass spectrometry-based M. tuberculosis identification and proteomics study. The results are important and useful for both basic and clinical M. tuberculosis studies.

## INTRODUCTION

Mycobacterium tuberculosis is the causative agent of tuberculosis (TB), which remains one of the world’s deadliest infectious killers, with nearly 1.4 million people succumbing to it in 2019 (1). Innovative strategies are needed to accelerate a safe and effective TB vaccine, improve responses to treatment, and introduce novel diagnostic approaches for earlier recognition of populations at risk. In addition, particular attention has been directed toward emerging cases of drug-resistant M. tuberculosis strains, which are arising due to the mismanagement of anti-TB treatments that amplify clinical strains with genetic mutations, especially in the promoter region (2). Molecular assays based on mass spectrometry analysis are recognized as emerging tools for rapid mycobacterial identification and drug susceptibility assessments (3–5). High-end techniques such as matrix-assisted laser desorption ionization–time of flight mass spectrometry (MALDI-TOF MS) and liquid chromatography-tandem MS (LC-MS/MS) have been applied for M. tuberculosis identification and proteomics profiling.

Drug-susceptible, drug-resistant, and multidrug-resistant M. tuberculosis strains are classified into biohazard risk group 3 based on the *WHO Laboratory Biosafety Manual*, as they pose a risk of serious human disease and can be readily transmitted. Extensively drug-resistant (XDR) M. tuberculosis strains were classified as risk group 4, capable of causing very serious human disease, with no effective treatment or preventive measures available yet (6). An extensive study of pathogenic bacteria requires at least a biosafety level 3 (BSL3) laboratory (7), which poses significant challenges in practice (8). Regions in developing countries with high TB burdens usually have limited resources for the recommended laboratory facilities. Indeed, several reports have raised concerns about laboratory-acquired TB infections, in which aerosols and skin punctures are the two major routes of transmission (9, 10).

Heat treatment is an attractive option for M. tuberculosis inactivation. While several groups have reported desirable inactivation efficiencies (11, 12), others have raised concerns that heat treatments cannot guarantee total microorganism inactivation (13–16) and thus may not be entirely safe. In addition, there is no consensus on specific protocols for heat inactivation of M. tuberculosis. Differences in temperature, container type, and duration of heating may result in inconsistencies among studies.

In addition to biosafety, any sample pretreatment procedure may affect the pathogen characteristics for downstream molecular analyses. Previous findings pointed out that 20 min of heat treatment at ∼80°C to 95°C inactivates M. tuberculosis while keeping the RNA intact (17), while another group incubated the microorganism at 96°C in 20% Chelex for 1 h and preserved the DNA (18). Still, both heat inactivation and chemical cell lysis procedures might cause denaturation of the protein and interfere with the results of subsequent proteomic analyses. To compare minor protein changes between different conditions, a suitable sample preparation process is required. However, there are scarce reports addressing the influence of bacterial inactivation on the efficacy of proteomics analyses for M. tuberculosis, which is actually an important issue to be investigated.

Since MS instruments are not available in every clinical laboratory, shipping of inactivated samples might be a choice after confirming the biosafety of the inactivation protocol. The present study aimed to evaluate the biosafety of different protocols for M. tuberculosis heat inactivation and observe whether protein components from the mycobacteria can be preserved for downstream MS-based bacterial identification and proteomics analyses.

## RESULTS

### Biosafety evaluation of heat inactivation methods.

**FIG 1:**
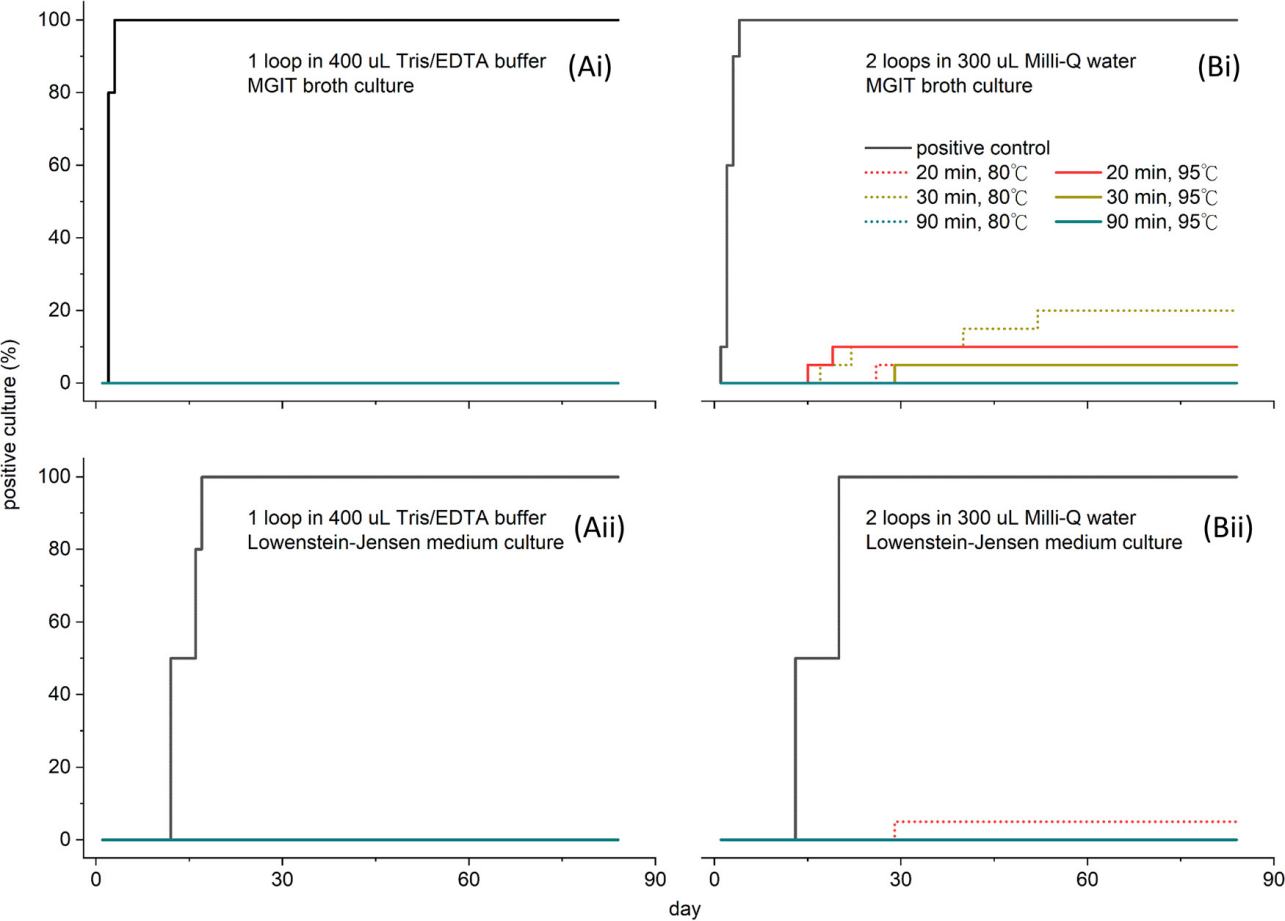
(Ai and Aii) Growth performances of the first set of samples subjected to the six inactivation processes. Growth evaluations were performed in both MGIT broth culture and LJ medium culture. (Bi and Bii) Growth performances of the second set of samples subjected to the six inactivation processes. Data are presented as the percent (%) of total colonies versus the day of observation. The total observation period is 84 days (12 weeks).

We conducted two sets of heat inactivation experiments with different M. tuberculosis biomass and solvent types. The first set was with one inoculation loop of biomass in Tris-EDTA (TE) buffer, and the second one used two loops of biomass in water. Six heat inactivation conditions were conducted for each set. Following six different heat inactivation protocols, no growth of M. tuberculosis was observed in either Lowenstein-Jensen (LJ) medium or mycobacterial growth indicator tubes (MGITs) for the first set, while the untreated positive control began to grow colonies on the third day of observation. Observations were performed for 12 weeks, with no apparent growth of bacteria (Fig. 1Ai and Aii). In contrast, some samples with two loops of biomass in water treated with a heating protocol of 80°C/20 min, 80°C/30 min, 95°C/20 min, or 95°C/30 min showed positive results during the growth test. Only the heat inactivation conducted for 90 min (80°C and 95°C) provided zero growth results (Fig. 1Bi and Bii).

### Effect of heat inactivation on MALDI-TOF MS bacterial identification.

**FIG 2:**
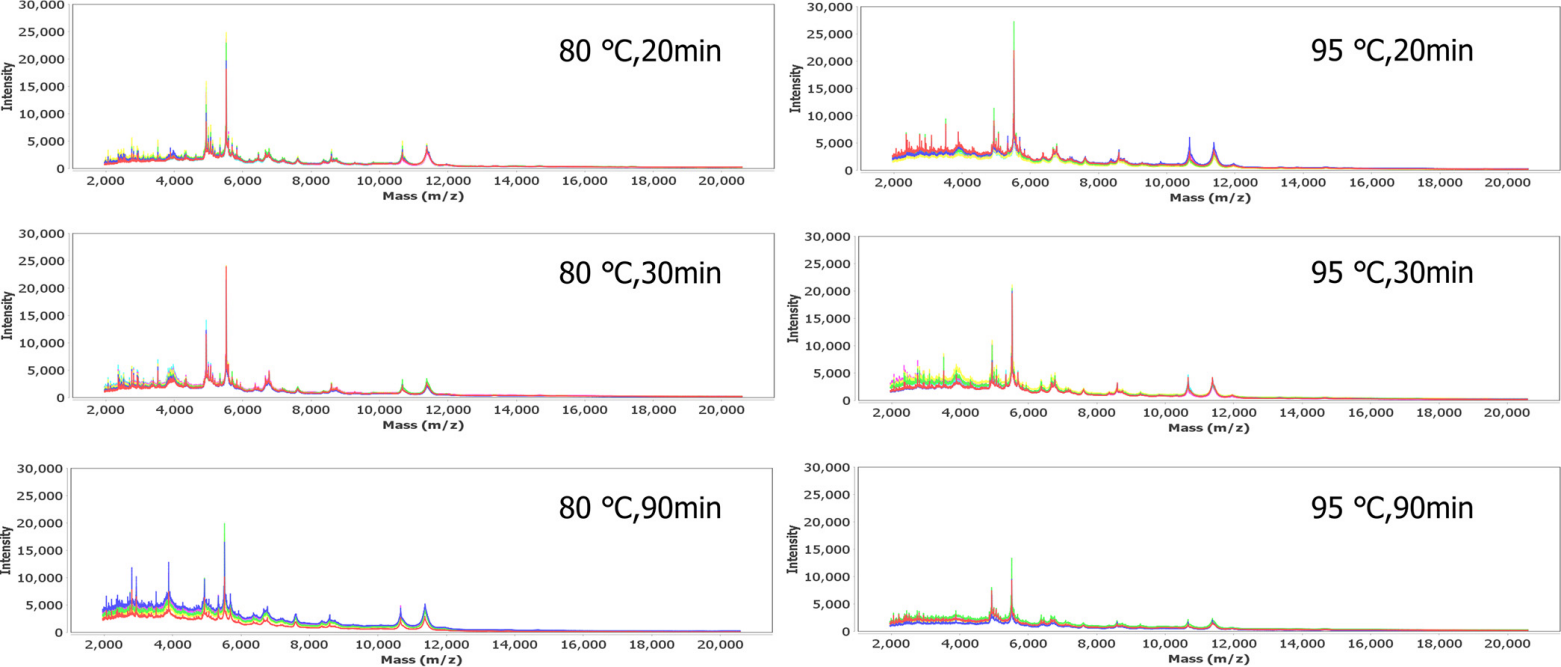
MALDI-TOF mass spectra of Mycobacterium tuberculosis (MTB) heat-inactivated samples treated with different temperatures and durations.

The results of the MALDI-TOF MS analysis for the purpose of M. tuberculosis identification following the heat inactivation process are shown in Table 1. Each treatment group yielded satisfactory outcomes (scores of ≥1.7) for M. tuberculosis identification; however, specimens that underwent the inactivation processes of 80°C/90 min and 95°C/30 min consistently had higher identification scores (>2.0). Although comparison of the whole-protein signals from MALDI-TOF MS spectra remained challenging, we observed that the intensities of signals with mass/charge ratios (*m/z*) of ∼2,000 to 5,000 were relatively higher for samples treated at 80°C/90 min, 95°C/20 min, and 95°C/30 min (Fig. 2). Accordingly, more sample numbers in these groups also showed scores of >2.0. We also noted that the overall intensities of peaks decreased in a manner dependent on the period of heat inactivation. This trend was observed in both the 80°C and 95°C groups.

**TABLE 1:** MALDI-TOF MS bacterial identification scores for M. tuberculosis specimens following the six heat inactivation processes

Inactivation temp (°C)	Inactivation duration (min)	No. of isolates tested	No. of isolates with a highest score of:		
			<1.7	1.7–2.0	≥2.0
80	20	6		2	4
80	30	6		4	2
80	90	6			6
95	20	6		1	5
95	30	6			6
95	90	6		3	3

**FIG 3:**
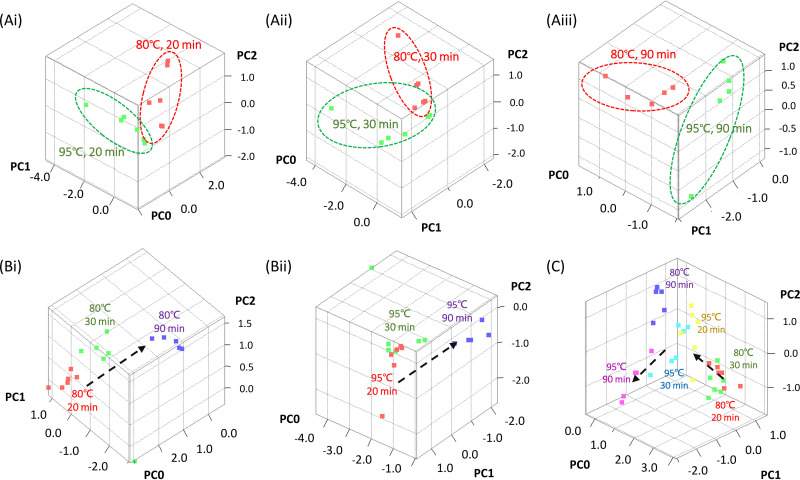
Principal-component analysis of MALDI-TOF results. (Ai to Aiii) Comparison of different heat inactivation temperatures. (Bi and Bii) Comparison of different heat inactivation times. (C) Comparison of the six heat inactivation conditions.

We further applied a principal-component analysis (PCA) to investigate clustering and the effects of different conditions on the MALDI-TOF MS analytical results. In Fig. 3Ai to Aiii, we observed that samples treated at different temperatures (80°C and 95°C) were aligned in two individual clusters despite having the same heating period. This result indicated that the 15°C difference in the heat inactivation temperatures resulted in variations of the compositions of the proteome profiles. The effects of the heating time can also be observed in Fig. 3Bi and Bii. The time trajectories indicated by the dashed arrows show that when we increased the heating time, there was a trend of profile changes resulting from protein composition. Clear clusters and a shifting of groups in the 3D PCA plot are also evident in Fig. 3C, which reveals that the protein profiles changed with a trend when we increased the temperature or heating time (dashed arrows). Interestingly, we identified that samples treated at 80°C/90 min, 95°C/20 min, and 95°C/30 min were clustered in the center of the 3D PCA plot, and these samples were also those with ID scores of >2.0.

### Proteome profiles with different heat inactivation protocols.

**FIG 4:**
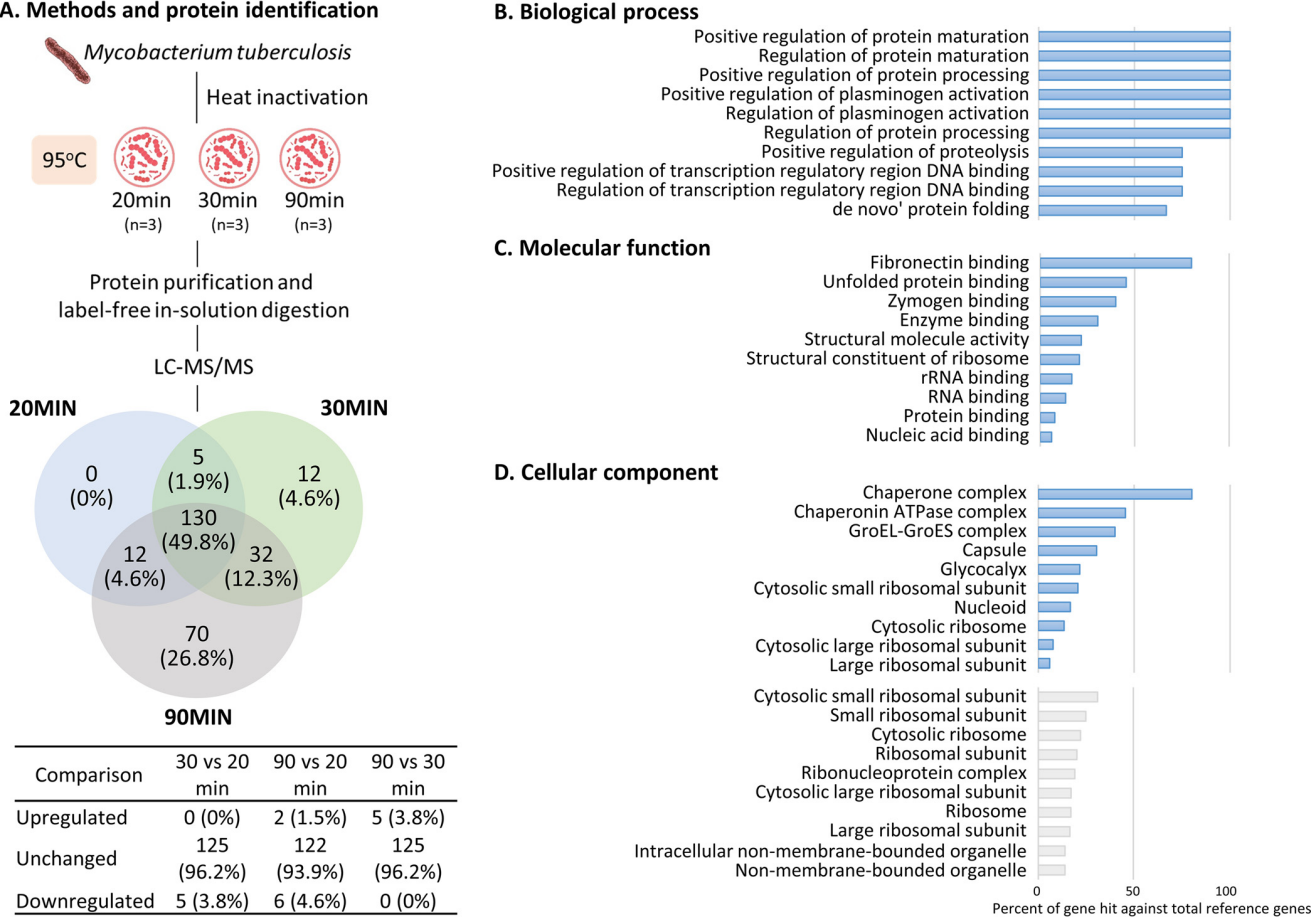
(A) Overlap of protein identification of three different heat inactivation methods and comparison of the common protein regulation. Upregulated and downregulated proteins were defined as ≥2 and ≤2 multiples of change, respectively. Annotation of (B) biological processes, (C) molecular functions, and (D) cellular components of the commonly quantified proteins (blue bars) and proteins uniquely quantified in the 90-min group (gray bars) by Gene Ontology analyses.

Total cell lysates from the three groups were collected for a label-free quantitative proteomics analysis. In the present study, 648 proteins were identified with confidence (*P < *0.05; protein false discovery rate [FDR], <1%). For quantification purposes, only proteins with at least two reported intensities from the three biological replicates were included, resulting in 147, 179, and 244 quantified proteins for the 20-, 30-, and 90-min groups, respectively. As presented in Fig. 4A, 130 proteins were commonly quantified in all three groups, 5 proteins were shared by the 20- and 30-min groups, 12 by the 20- and 90-min groups, and 32 by the 30- and 90-min groups. In addition, 12 and 70 proteins were uniquely quantified in the 30- and 90-min groups, respectively. Among the 130 common proteins, comparison analysis revealed over 93% consistency among all three groups. Proteins with inconsistent expression (with a multiple of change of >2-fold) between groups are listed in Table 2. To note, the alanine- and proline-rich secreted protein (APA) was found at a higher intensity in the 90-min group, while the trehalose monomycolate exporter (MMPL3) was found at a lower intensity in the 20-min group. APA is a potent antigen in animals immunized with live bacteria (19) which was shown to elicit strong delayed-type hypersensitivity and a type 1 T-cell response in healthy humans (20); MMPL3 functions as an important lipid transporter across inner membranes and is a target of the antitubercular drug SQ109 (21, 22).

**TABLE 2:** List of proteins with up- and downregulated expression among different heat inactivation periods

Protein ID	Protein name	No. of peptides	No. of razor + unique peptides	No. of unique peptides	Sequence coverage (%)	Score	Mean LFQ^*a*^ intensity at:			Fold change		
							20 min	30 min	90 min	30 vs 20 min	90 vs 20 min	90 vs 30 min
tr|I6XY36|I6XY36_MYCTU	Conserved protein	3	3	3	59	75	1,575,300	2,104,567	4,835,233	→1.34^*b*^	↑3.07	↑2.30
sp|P9WG55|TIG_MYCTU	Trigger factor	12	12	12	41	207	15,561,567	5,889,300	12,313,733	↓0.38	→0.79	↑2.09
tr|I6WZM9|I6WZM9_MYCTU	Probable cold shock-like protein B CspB	6	6	6	44	47	12,449,600	5,736,633	11,808,367	↓0.46	→0.95	↑2.06
sp|P9WIR7|APA_MYCTU	Alanine and proline-rich secreted protein Apa	10	10	10	36	102	15,094,733	11,045,967	22,272,000	→0.73	→1.48	↑2.02
sp|O06291|HTRA1_MYCTU	Probable serine protease HtrA1	6	6	6	17	37	4,271,000	2,615,200	5,256,267	→0.61	→1.23	↑2.01
sp|P9WHA3|RL30_MYCTU	50S ribosomal protein L30	2	2	2	43	105	8,095,500	14,713,333	16,803,567	→1.82	↑2.08	→1.14
sp|P9WJV5|MMPL3_MYCTU	Trehalose monomycolate exporter MmpL3	8	8	8	14	77	6,792,050	2,879,250	2,572,700	↓0.42	↓0.38	→0.89
sp|P9WGA1|TATA_MYCTU	Sec-independent protein translocase protein TatA	2	2	2	47	78	16,745,000	7,532,333	6,483,200	↓0.45	↓0.39	→0.86
sp|P9WNI1|ESXC_MYCTU	ESAT-6-like protein EsxC	1	1	1	22	38	8,099,300	3,913,800	3,315,467	↓0.48	↓0.41	→0.85
sp|P9WG35|TPX_MYCTU	Thiol peroxidase	7	7	7	72	89	1,477,050	920,855	624,297	→0.62	↓0.42	→0.68
tr|I6XWF9|I6XWF9_MYCTU	Uncharacterized protein	2	2	2	57	7	3,045,000	1,714,233	1,145,833	→0.56	↓0.38	→0.67
sp|Q6MX43|DODEC_MYCTU	Calcium dodecin	3	3	3	61	29	4,225,133	2,637,267	1,666,667	→0.62	↓0.39	→0.63

a LFQ, label-free quantification.

b Up- and down-regulated proteins are defined as fold-change of ≥2 (upward arrow) and ≤-2 (downward arrow), respectively.

The Gene Ontology (GO) analysis identified commonly quantified proteins enriched in dysregulated biological processes, molecular functions, and cellular components and pathways. The top 10 enriched biological processes included the regulation of protein maturation and processing, plasminogen activation, transcription of DNA binding, and proteolysis (Fig. 4B). Enriched molecular functions included the binding of fibronectin, unfolded proteins, zymogen, enzymes, nucleic acids, RNA, and proteins (Fig. 4C). Common proteins from the three groups were identified as being part of the chaperone complex, capsule, nucleoid, and (large) cytosolic ribosome (Fig. 4D, blue bars). We further analyzed the proteins uniquely quantified in the 90-min group (Table S1) and identified localization within the (small) ribosomal subunit, ribonucleoprotein complex, and non-membrane-bound organelles (Fig. 4D, gray bars).

## DISCUSSION

In this study, two sets of heat inactivation experiments were conducted for biosafety evaluation. In the first set, an inoculation loop of biomass submerged in TE buffer was used; no mycobacterial growth was observed for any of the six heat inactivation conditions. In the second set, two loops of biomass in a water solvent were used to mimic the condition used for mass spectrometry study, and we found that a longer heating time was required for optimal inactivation. We speculated that the higher mycobacterial burden within the sample solution may form aggregation, thus resulting in bigger particles of biomass, and 20 to 30 min of heating was insufficient to inactivate all mycobacteria. Previous studies also pointed out that the pH value of the solution used for mycobacterial samples affects their surface charges and zeta potential (23, 24). When the pH is lower, the negative charges of the mycobacterial surface decreases, and aggregation of mycobacterial particles occurs. Compared to the TE buffer (pH 8), pure water has a lower pH value of less than 7. This might also be one of the reasons that the two loops of biomass in water needed a longer heat inactivation time.

As a pathogenic organism posing biosafety threats, the manipulation and study of M. tuberculosis require BSL3 laboratory operational and physical protective measures (25), which might not be readily accessible in the rural areas of developing countries, where the TB burden is high. Therefore, a reliable method of mycobacterial inactivation is expected to provide biosafety assurance for laboratory personnel and promote TB research. With a reliable and easy inactivation process, the samples can be inactivated before being sent to a laboratory with high-end instruments such as mass spectrometers, which benefits bacterial identification and research.

Previous studies adopted a water bath heating method for M. tuberculosis inactivation for 20 min at 80°C (12, 17) and reported excellent biosafety, while others failed to show complete inactivation with the same protocol (13, 15). A study that applied specimen drying on a thermal block reported that the procedure was insufficient to completely inactivate M. tuberculosis (26). To minimize the possibility of sporadic growth resulting from inefficient heating due to a large bacterial load or high-density cultures (12, 14, 15), we maintained a total bacterial suspension volume of <500 μL inside a 1.5-mL microtube, ensuring adequate space to expose every part of the sample to heating.

From the literature survey, 20 min of inactivation at 80°C was found to preserve a sufficient amount of DNA and RNA of M. tuberculosis for downstream molecular tests (12, 17, 18). However, previous studies have not addressed the effects of heat inactivation and sample preparation on analysis of the protein profile by using MALDI-TOF MS and LC-MS/MS. In this study, we adopted a sample preparation method with silicate bead beating after heat inactivation. Mycobacteria are well known to have a thick waxy outer cell wall. It is composed of mycolic acids and arabinogalactans which associate with the envelope consists peptidoglycan. General lysis methods are therefore inefficient for M. tuberculosis disruption, and a harsh technique is sometimes required (27). Silicate bead beating was reported to enhance disruption of the cell wall structure and improve protein harvests (5, 27). A previous study evaluated an extraction method adopting 30 min of heat inactivation at ∼95°C to 100°C in a heating block, followed by the addition of 100% ethanol and zirconia/silica beads in acetonitrile, which showed comparable MALDI-TOF MS identification scores for M. tuberculosis and nontuberculosis Mycobacterium species as a conventional extraction method (5). Applying a similar extraction protocol, we evaluated distinct heat inactivation methods and showed that each method gave satisfying identification scores (>1.7). Among the six conditions, heating at 80°C for 90 min and at 95°C for 30 min consistently produced higher identification scores (>2.0) and similar MALDI-TOF MS profiles using PCA analysis.

For LC-MS/MS proteomics analysis, prior studies utilized combinations of lysis buffers, beads, and a sonication step to break open mycobacterial cells and extract proteins. However, these studies mostly focused on the protein profiling and identification without further accounting for the biosafety properties of the approach (28, 29). In the current study, we showed that heat inactivation treatment produced satisfactory protein preservation for downstream proteomics assays. Heating at 95°C for 90 min resulted in higher numbers of identified proteins compared to shorter time periods. This observation indicates that the loss of proteins is most likely not due to heat-induced protein degradation and supports similar observations of RNA abundances (17). In response to stress, a buildup of unfinished ribosomes may be present in the nuclei, termed the “ribosome assembly stress response” (30). Therefore, the unique “accumulation” of ribosomal proteins found with 90 min of treatment may be a result of the heat stress response. Another possible hypothesis is that the ribosome component itself may be relatively stable under heat stress, as was reported in a heat-resistant strain of Escherichia coli (31).

Our study identified a relatively lower number of proteins following the LC/MS-MS assay in all three groups, compared to previous findings (28, 32, 33), which may have resulted from the lack of a prefractionation approach to reduce the sample complexity (34). Generally, this step requires a sufficient amount of material, and the selection of an appropriate method depends on the study purpose. For in-depth protein characterization of M. tuberculosis, a more rigorous method should be employed to improve the identification and quantification of more peptides and proteins, including those with lower abundances. In addition, we only examined the laboratory-grown strain H37Rv. Future studies should aim to observe the biosafety of the heat inactivation method and protein preservation efficiency for other M. tuberculosis strains, including clinical specimens.

In summary, we report an approach for inactivating M. tuberculosis by applying heat in a thermal block. All 6 heat inactivation conditions resulted in the absence of growth for samples with 1 inoculation loop of biomass in TE buffer. Heating at both 80°C and 95°C for 90 min resulted in the absence of bacterial growth for samples with 2 loops of biomass in water. Further investigation indicated that heat inactivation of M. tuberculosis at either 80°C for 90 min or 95°C for 30 min was efficient for species identification using MALDI-TOF MS assays. A high-throughput LC/MS-MS analysis further revealed comparable protein expression among three different heating periods, confirming that the heat inactivation protocol is safe and reliable for preserving the protein components of mycobacteria. Nevertheless, all mycobacterial inactivation conditions should be evaluated in the laboratory to validate the in-house biosafety practices of handling mycobacterial materials.

## MATERIALS AND METHODS

### Study site.

This study was carried out at a biosafety level P2 plus TB laboratory at Wan Fang Hospital, Taipei Medical University (Taipei, Taiwan). The laboratory received accreditation from the College of American Pathologists Laboratory Accreditation Program and the Taiwan Accreditation Foundation and is authorized by the Taiwan Centers for Disease Control to conduct mycobacterial examinations in northern Taiwan.

### Specimens and heat inactivation methods.

In total, 260 specimens of M. tuberculosis strain H37Rv were collected for the study. The specimens were cultured in Middlebrook 7H11 medium supplemented with 2% (vol/vol) glycerol, 0.5% (vol/vol) Tween 80, and 10% (vol/vol) oleic acid-albumin-dextrose-catalase (OADC) at 37°C for ∼10 to 14 days prior to the experiment. Two sets of heat inactivation experiments were conducted for evaluation. For the first set, an inoculation loop of biomass from fresh cultures was harvested and placed into a snap cap 1.5-mL microcentrifuge tube (SMB-M1540-C; New Taipei City, Taiwan) containing 400 μL Tris/EDTA buffer (pH 8.0; Thermo Scientific Pierce, Rockford, IL, USA), tightly closed for heating. Of the 130 specimens, 10 were kept as a positive growth control, and 120 were randomly assigned to six different heat inactivation methods (with 20 samples per group), in a dry heat thermal block (CLUBIO CB-1501 dry bath; Taiwan Xinqi Co., Ltd., Taipei, Taiwan): (i) 80°C for 20 min; (ii) 80°C for 30 min; (iii) 80°C for 90 min; (iv) 95°C for 20 min; (v) 95°C for 30 min; and (vi) 95°C for 90 min. The tubes were left to stand for 10 min to allow any aerosols to settle prior to opening. For the second set of experiments, samples were prepared by placing two inoculation loops of biomass into a microcentrifuge tube with 300 μL Milli-Q (MQ) water. The control and heat inactivation groups were assigned as for the first set.

### Confirmation of bacterial inactivation (biosafety evaluation).

To assess the inactivation efficacy, aliquots of 100 and 200 μL from each sample were collected for respective inoculation in LJ medium and in MGITs (Becton, Dickinson, Sparks, MD, USA). Cultures in LJ medium were kept at appropriate incubation conditions (37°C, 10% CO_2_; Thermo Fisher Scientific Forma 3950 CO_2_ Incubator; Marietta, OH, USA), while the MGITs were incubated in a Bactec MGIT 960 instrument (Becton, Dickinson). All samples were examined for bacterial growth daily during the first week and weekly thereafter, for a total of 12 weeks. In the positive cultures, Ziehl-Neelsen staining was performed to confirm the presence of acid-fast bacilli.

### Bacterial identification by MALDI-TOF MS.

#### (i) Sample preparation for MALDI-TOF MS.

For species identification with MALDI-TOF MS, we introduced samples from two loopfuls of freshly cultured M. tuberculosis H37Rv into a 1.5-mL microtube (SMB-M1540-C) containing 300 μL water. The samples were inactivated as described above using the six heat inactivation methods, 80°C/20 min, 80°C/30 min, 80°C/90 min, 95°C/20 min, 95°C/30 min, and 95°C/90 min. For each group, six biological replicates were prepared. The following protocol was performed according to the working protocol from Bruker Daltonics (Bremen, Germany) to identify the mycobacterial isolates (revised 2 January 2013).

Briefly, after removing the supernatant by centrifugation at 13,000 rpm for 2 min, 50 μL water was added to the pellet, and the microtubes were tightly closed and heated for another 10 min at 95°C in a thermal block (CLUBIO CB-1501 dry bath). The sample was left to cool prior to opening the cap, 1.2 mL of cold 100% alcohol (previously stored in a freezer at approximately −18°C to −20°C) was added, and the mixture was centrifuged at 13,000 rpm for 2 min. Subsequently, we removed all of the supernatant and left the pellet to dry for 5 min with the tube open to allow the ethanol to completely evaporate. Using the tip of a small spatula, we added silica beads (0.5-mm zirconia/silica beads) and 20 μL pure acetonitrile and mixed the samples well for 20 min by vortexing. We then added 20 μL 70% formic acid and centrifuged this at 13,000 rpm for 2 min. From each sample, 1 μL supernatant was placed in each of the 96 spots of an MSP96 polished steel target plate (Bruker Daltonics), and these were allowed to dry at room temperature. Finally, 1 μL HCCA matrix solution (α-cyano-4-hydroxycinnamic acid) was added to each spot and left to crystallize at room temperature before further analysis by MALDI-TOF MS.

#### (ii) MALDI-TOF MS analysis.

We adapted the Microflex LT MALDI-TOF MS (Bruker Daltonics) to acquire the spectra in the linear positive-ion mode (with a laser frequency of 60 Hz and a mass/charge ratio [*m*/*z*] of ∼2 to 2,000 Da). The protein profile was respectively obtained and analyzed using FlexControl v3.4 and FlexAnalysis v3.4 software (both from Bruker Daltonics). Mycobacteria Library v2.0 was used; it contains 313 mycobacterial protein profiles, representing 131 species.

The quality of spectra generated by the mycobacterial extraction protocol for MALDI-TOF MS was evaluated according to the following criteria. The software assigned a score of ∼0 to 3 and classified the results into three categories: reliable (species level; ≥2), probable (genus level; ∼1.7 to 1.999), and nonidentifiable (<1.7). Identification was proven when a score of ≥1.70 was obtained and when the identification matched at least 5 of the top 10 species identifications provided by MALDI-TOF MS. We considered a range of ∼2.0 to 3.0 as acceptable, and scores of ∼1.7 to 2.0 were considered consistent when the same identification was repeated in most of the 10 possibilities provided by the project. Lower scores (<1.7) were reported as unreliable identification (35–37).

### Label-free proteomics analysis.

#### (i) Protein extraction and digestion.

For the proteomics analysis, we selected three heat inactivation methods, 95°C/20 min, 95°C/30 min, and 95°C/90 min, with three biological replicates per group. We adapted the modified inactivated mycobacteria bead preparation method (Bruker Daltonics) as described below. Following heat inactivation, the samples were centrifuged (13,000 rpm for 2 min; tabletop microcentrifuge model 3300; Kubota, Japan), and the supernatant was removed. A new 50-μL fraction of water was added, and the sample was again heated to 95°C for 10 min. After cooling, 1,200 μL cold ethanol was added with a short period of mixing. The supernatant was removed after centrifuging the sample at 13,000 relative centrifugal force (RCF) for 2 min. Silica beads (0.5 mm) and 20 μL acetonitrile were added, and the sample was vortex mixed for 20 min. The supernatant was collected and quantified using a Pierce bicinchoninic acid (BCA) assay (Thermo Fisher Scientific, Rockford, IL, USA) to determine the protein concentration. Thirty micrograms of protein was subjected to label-free in-solution digestion. Briefly, 200 μL ammonium bicarbonate (50 mM) buffer with a protease inhibitor (Amresco, Solon, OH, USA) was added to each sample. For protein reduction, 4 μL dithiothreitol (550 mM) was added, and the sample was heated to 56°C for 45 min. Iodoacetamide (8 μL, 450 mM) was then added, and the sample was incubated at room temperature in the dark for 45 min. Protein digestion was conducted by adding sequencing-grade trypsin, and the sample was incubated at 37°C overnight. The reaction was finally stopped by adding 20 μL of 10% formic acid. All samples were desalted using a C18 solid-phase extraction cartridge (Sep-Pak C18 1-cc cartridge; Waters, Dublin, Ireland) prior to the LC-MS/MS analysis.

#### (ii) LC-MS/MS analysis.

The recovered peptides were diluted in 0.1% formic acid (buffer A) and loaded onto a reverse-phase column (Zorbax 300SB-C18, 0.3 × 5 mm; Agilent Technologies, Santa Clara, CA, USA) for the high-performance LC (HPLC) step. Subsequently, we separated the desalted peptides using a homemade column (HydroRP 2.5 μm, 75 μm inner diameter [i.d.] × 20 cm with a 15-μm tip), with a multistep gradient of 99.9% acetonitrile/0.1% formic acid (buffer B) solution for 70 min at a flow rate of 0.3 μL/min. The LC system was coupled to the 2D linear ion trap MS (Orbitrap Elite ETD; Thermo Fisher). MS data were acquired using Xcalibur v2.2 software (Thermo Fisher). The full-scan MS was performed over a range of ∼400 to 2,000 Da, and the resolution was set at 120,000 at *m*/*z* 400. We initiated internal calibration by setting the ion signal of protonated dodecamethylcyclohexasiloxane ions to *m/z* 536.165365 as the lock mass. An additional MS scan was performed for the 12 most abundant precursor ions after obtaining the 12 data-dependent MS/MS scan events in the preview MS scan. The *m/z* values selected for MS/MS were dynamically excluded for 60 s with a relative mass window of 15 ppm. The electrospray voltage was set to 2.0 kv and the capillary temperature to 200°C. MS and MS/MS automatic gain control were fixed at 1,000 ms (full scan) and 300 ms (MS/MS) or 3 × 10^6^ ions (full scan) and 3 × 10^4^ ions (MS/MS) for the maximum accumulated time or ions, respectively.

#### (iii) Protein identification and quantification.

The raw data from the LC-MS/MS analysis were analyzed using MaxQuant v1.6.7.0 software (http://maxquant.org/) (38) and searched against the UniProt Mycobacterium tuberculosis H37Rv FASTA databases (3,993 total entries, downloaded on 2 February 21) using the built-in Andromeda search engine (39). The following search parameters were set: trypsin with full specificity and a maximum of two missed cleavages for enzyme properties, a precursor mass window of 6 ppm, a precursor mass tolerance of 20 ppm, fixed modification of carbamidomethylation (C) of cysteines, variable modifications of methionine oxidation, and protein N-terminal acetylation. Stringent criteria for peptide-spectrum match (PSM) and a protein FDR of 1% were applied. Hits that were identified only by site, found in decoy or contaminant lists, or identified with fewer than two peptides were excluded. Label-free quantification was performed with MaxQuant software as previously described (40). Protein abundances were calculated using the normalized spectral protein intensity (label-free quantification [LFQ] intensity). Only proteins identified in at least two samples were included in the analysis. Statistical analyses that compared the difference between each heat inactivation time group were performed using an unpaired *t* test.

#### (iv) Functional enrichment analysis.

Quantified proteins were subjected to functional analyses using Gene Ontology (GO; release 1 June 2019) (41, 42) for annotation of the biological processes, molecular functions, and cellular components. Annotations with *P < *0.05 were considered significant.

### Data availability.

The mass spectrometry proteomics data were deposited at the ProteomeXchange Consortium via the PRIDE (43) partner repository under the data set identifier PXD027011.

## References

[1] Reid MJA, Arinaminpathy N, Bloom A, Bloom BR, Boehme C, Chaisson R, Chin DP, Churchyard G, Cox H, Ditiu L, Dybul M, Farrar J, Fauci AS, Fekadu E, Fujiwara PI, Hallett TB, Hanson CL, Harrington M, Herbert N, Hopewell PC, Ikeda C, Jamison DT, Khan AJ, Koek I, Krishnan N, Motsoaledi A, Pai M, Raviglione MC, Sharman A, Small PM, Swaminathan S, Temesgen Z, Vassall A, Venkatesan N, van Weezenbeek K, Yamey G, Agins BD, Alexandru S, Andrews JR, Beyeler N, Bivol S, Brigden G, Cattamanchi A, Cazabon D, Crudu V, Daftary A, Dewan P, Doepel LK, Eisinger RW, Fan V, et al. 2019. Building a tuberculosis-free world: the Lancet Commission on tuberculosis. Lancet 393:1331–1384. doi:10.1016/S0140-6736(19)30024-8. 30904263

[2] Smith T, Wolff KA, Nguyen L. 2013. Molecular biology of drug resistance in Mycobacterium tuberculosis. Curr Top Microbiol Immunol 374:53–80. doi:10.1007/82_2012_279. 23179675 PMC3982203

[3] Bespyatykh JA, Shitikov EA, Ilina EN. 2017. Proteomics for the investigation of mycobacteria. Acta Naturae 9:15–25. doi:10.32607/20758251-2017-9-1-15-25. 28461970 PMC5406656

[4] Banaei-Esfahani A, Nicod C, Aebersold R, Collins BC. 2017. Systems proteomics approaches to study bacterial pathogens: application to Mycobacterium tuberculosis. Curr Opin Microbiol 39:64–72. doi:10.1016/j.mib.2017.09.013. 29032348 PMC5732070

[5] Adams LL, Salee P, Dionne K, Carroll K, Parrish N. 2015. A novel protein extraction method for identification of mycobacteria using MALDI-ToF MS. J Microbiol Methods 119:1–3. doi:10.1016/j.mimet.2015.09.010. 26392293

[6] Xu Y, Wang G, Xu M. 2020. Biohazard levels and biosafety protection for Mycobacterium tuberculosis strains with different virulence. Biosaf Health 2:135–141. doi:10.1016/j.bsheal.2020.04.001.

[7] Furin J, Cox H, Pai M. 2019. Tuberculosis. Lancet 393:1642–1656. doi:10.1016/S0140-6736(19)30308-3. 30904262

[8] Ssengooba W, Gelderbloem SJ, Mboowa G, Wajja A, Namaganda C, Musoke P, Mayanja-Kizza H, Joloba ML. 2015. Feasibility of establishing a biosafety level 3 tuberculosis culture laboratory of acceptable quality standards in a resource-limited setting: an experience from Uganda. Health Res Policy Sys 13:4. doi:10.1186/1478-4505-13-4. PMC4326287 25589057

[9] Kao AS, Ashford DA, McNeil MM, Warren NG, Good RC. 1997. Descriptive profile of tuberculin skin testing programs and laboratory-acquired tuberculosis infections in public health laboratories. J Clin Microbiol 35:1847–1851. doi:10.1128/jcm.35.7.1847-1851.1997. 9196206 PMC229854

[10] Menzies D, Fanning A, Yuan L, Fitzgerald M. 1995. Tuberculosis among health care workers. N Engl J Med 332:92–98. doi:10.1056/NEJM199501123320206. 7990907

[11] Castro C, Gonzalez L, Rozo JC, Puerto G, Ribon W. 2009. Biosafety evaluation of the DNA extraction protocol for Mycobacterium tuberculosis complex species, as implemented at the Instituto Nacional de Salud, Colombia. Biomedica 29:561–566. 20440455

[12] Doig C, Seagar AL, Watt B, Forbes KJ. 2002. The efficacy of the heat killing of Mycobacterium tuberculosis. J Clin Pathol 55:778–779. doi:10.1136/jcp.55.10.778. 12354807 PMC1769777

[13] Bemer-Melchior P, Drugeon HB. 1999. Inactivation of Mycobacterium tuberculosis for DNA typing analysis. J Clin Microbiol 37:2350–2351. doi:10.1128/JCM.37.7.2350-2351.1999. 10364613 PMC85159

[14] Blackwood KS, Burdz TV, Turenne CY, Sharma MK, Kabani AM, Wolfe JN. 2005. Viability testing of material derived from Mycobacterium tuberculosis prior to removal from a containment level-III laboratory as part of a laboratory risk assessment program. BMC Infect Dis 5:4. doi:10.1186/1471-2334-5-4. 15667662 PMC548516

[15] Somerville W, Thibert L, Schwartzman K, Behr MA. 2005. Extraction of Mycobacterium tuberculosis DNA: a question of containment. J Clin Microbiol 43:2996–2997. doi:10.1128/JCM.43.6.2996-2997.2005. 15956443 PMC1151963

[16] Zwadyk P, Jr, Down JA, Myers N, Dey MS. 1994. Rendering of mycobacteria safe for molecular diagnostic studies and development of a lysis method for strand displacement amplification and PCR. J Clin Microbiol 32:2140–2146. doi:10.1128/jcm.32.9.2140-2146.1994. 7814537 PMC263956

[17] Sabiiti W, Azam K, Esmeraldo E, Bhatt N, Rachow A, Gillespie SH. 2019. Heat inactivation renders sputum safe and preserves Mycobacterium tuberculosis RNA for downstream molecular tests. J Clin Microbiol 57:e01778-18. doi:10.1128/JCM.01778-18. 30728191 PMC6440770

[18] Djelouagji Z, Drancourt M. 2006. Inactivation of cultured Mycobacterium tuberculosis organisms prior to DNA extraction. J Clin Microbiol 44:1594–1595. doi:10.1128/JCM.44.4.1594-1595.2006. 16597905 PMC1448686

[19] Romain F, Horn C, Pescher P, Namane A, Riviere M, Puzo G, Barzu O, Marchal G. 1999. Deglycosylation of the 45/47-kilodalton antigen complex of Mycobacterium tuberculosis decreases its capacity to elicit in vivo or in vitro cellular immune responses. Infect Immun 67:5567–5572. doi:10.1128/IAI.67.11.5567-5572.1999. 10531201 PMC96927

[20] Kumar P, Amara RR, Challu VK, Chadda VK, Satchidanandam V. 2003. The Apa protein of Mycobacterium tuberculosis stimulates gamma interferon-secreting CD4+ and CD8+ T cells from purified protein derivative-positive individuals and affords protection in a guinea pig model. Infect Immun 71:1929–1937. doi:10.1128/IAI.71.4.1929-1937.2003. 12654810 PMC152084

[21] Grzegorzewicz AE, Pham H, Gundi VA, Scherman MS, North EJ, Hess T, Jones V, Gruppo V, Born SE, Kordulakova J, Chavadi SS, Morisseau C, Lenaerts AJ, Lee RE, McNeil MR, Jackson M. 2012. Inhibition of mycolic acid transport across the Mycobacterium tuberculosis plasma membrane. Nat Chem Biol 8:334–341. doi:10.1038/nchembio.794. 22344175 PMC3307863

[22] Tahlan K, Wilson R, Kastrinsky DB, Arora K, Nair V, Fischer E, Barnes SW, Walker JR, Alland D, Barry CE, III, Boshoff HI. 2012. SQ109 targets MmpL3, a membrane transporter of trehalose monomycolate involved in mycolic acid donation to the cell wall core of Mycobacterium tuberculosis. Antimicrob Agents Chemother 56:1797–1809. doi:10.1128/AAC.05708-11. 22252828 PMC3318387

[23] Kłodzińska E, Szumski M, Dziubakiewicz E, Hrynkiewicz K, Skwarek E, Janusz W, Buszewski B. 2010. Effect of zeta potential value on bacterial behavior during electrophoretic separation. Electrophoresis 31:1590–1596. doi:10.1002/elps.200900559. 20422634

[24] Ayala-Torres C, Hernández N, Galeano A, Novoa-Aponte L, Soto C-Y. 2014. Zeta potential as a measure of the surface charge of mycobacterial cells. Ann Microbiol 64:1189–1195. doi:10.1007/s13213-013-0758-y.

[25] Department of Health and Human Services, Public Health Service, Centers for Disease Control and Prevention, National Institutes of Health. 1995. Primary containment for biohazards: selection, installation and use of biological safety cabinets. U.S. Government Printing Office, Washington, DC, USA.

[26] Chedore P, Th'ng C, Nolan DH, Churchwell GM, Sieffert DE, Hale YM, Jamieson F. 2002. Method for inactivating and fixing unstained smear preparations of mycobacterium tuberculosis for improved laboratory safety. J Clin Microbiol 40:4077–4080. doi:10.1128/JCM.40.11.4077-4080.2002. 12409378 PMC139704

[27] Rabodoarivelo MS, Aerts M, Vandamme P, Palomino JC, Rasolofo V, Martin A. 2016. Optimizing of a protein extraction method for Mycobacterium tuberculosis proteome analysis using mass spectrometry. J Microbiol Methods 131:144–147. doi:10.1016/j.mimet.2016.10.021. 27984057

[28] Jhingan GD, Kumari S, Jamwal SV, Kalam H, Arora D, Jain N, Kumaar LK, Samal A, Rao KVS, Kumar D, Nandicoori VK. 2016. Comparative proteomic analyses of avirulent, virulent, and clinical strains of Mycobacterium tuberculosis identify strain-specific patterns. J Biol Chem 291:14257–14273. doi:10.1074/jbc.M115.666123. 27151218 PMC4933181

[29] Yuan P, He L, Chen D, Sun Y, Ge Z, Shen D, Lu Y. 2020. Proteomic characterization of Mycobacterium tuberculosis reveals potential targets of bostrycin. J Proteomics 212:103576. doi:10.1016/j.jprot.2019.103576. 31706025

[30] Albert B, Kos-Braun IC, Henras AK, Dez C, Rueda MP, Zhang X, Gadal O, Kos M, Shore D. 2019. A ribosome assembly stress response regulates transcription to maintain proteome homeostasis. Elife 8:e45002. doi:10.7554/eLife.45002. 31124783 PMC6579557

[31] Pleitner A, Zhai Y, Winter R, Ruan L, McMullen LM, Ganzle MG. 2012. Compatible solutes contribute to heat resistance and ribosome stability in Escherichia coli AW1.7. Biochim Biophys Acta 1824:1351–1357. doi:10.1016/j.bbapap.2012.07.007. 22841996

[32] Malen H, De Souza GA, Pathak S, Softeland T, Wiker HG. 2011. Comparison of membrane proteins of Mycobacterium tuberculosis H37Rv and H37Ra strains. BMC Microbiol 11:18. doi:10.1186/1471-2180-11-18. 21261938 PMC3033788

[33] Schubert OT, Ludwig C, Kogadeeva M, Zimmermann M, Rosenberger G, Gengenbacher M, Gillet LC, Collins BC, Rost HL, Kaufmann SH, Sauer U, Aebersold R. 2015. Absolute proteome composition and dynamics during dormancy and resuscitation of Mycobacterium tuberculosis. Cell Host Microbe 18:96–108. doi:10.1016/j.chom.2015.06.001. 26094805

[34] Gengenbacher M, Mouritsen J, Schubert OT, Aebersold R, Kaufmann SHE. 2014. Mycobacterium tuberculosis in the proteomics era. Microbiol Spectr 2:2.2.05. doi:10.1128/microbiolspec.MGM2-0020-2013. 26105825

[35] Barberis C, Almuzara M, Join-Lambert O, Ramirez MS, Famiglietti A, Vay C. 2014. Comparison of the Bruker MALDI-TOF mass spectrometry system and conventional phenotypic methods for identification of Gram-positive rods. PLoS One 9:e106303. doi:10.1371/journal.pone.0106303. 25184254 PMC4153636

[36] Jamal WY, Ahmad S, Khan ZU, Rotimi VO. 2014. Comparative evaluation of two matrix-assisted laser desorption/ionization time-of-flight mass spectrometry (MALDI-TOF MS) systems for the identification of clinically significant yeasts. Int J Infect Dis 26:167–170. doi:10.1016/j.ijid.2014.05.031. 25080355

[37] Saleeb PG, Drake SK, Murray PR, Zelazny AM. 2011. Identification of mycobacteria in solid-culture media by matrix-assisted laser desorption ionization-time of flight mass spectrometry. J Clin Microbiol 49:1790–1794. doi:10.1128/JCM.02135-10. 21411597 PMC3122647

[38] Cox J, Matic I, Hilger M, Nagaraj N, Selbach M, Olsen JV, Mann M. 2009. A practical guide to the MaxQuant computational platform for SILAC-based quantitative proteomics. Nat Protoc 4:698–705. doi:10.1038/nprot.2009.36. 19373234

[39] Tyanova S, Temu T, Cox J. 2016. The MaxQuant computational platform for mass spectrometry-based shotgun proteomics. Nat Protoc 11:2301–2319. doi:10.1038/nprot.2016.136. 27809316

[40] Cox J, Neuhauser N, Michalski A, Scheltema RA, Olsen JV, Mann M. 2011. Andromeda: a peptide search engine integrated into the MaxQuant environment. J Proteome Res 10:1794–1805. doi:10.1021/pr101065j. 21254760

[41] Ashburner M, Ball CA, Blake JA, Botstein D, Butler H, Cherry JM, Davis AP, Dolinski K, Dwight SS, Eppig JT, Harris MA, Hill DP, Issel-Tarver L, Kasarskis A, Lewis S, Matese JC, Richardson JE, Ringwald M, Rubin GM, Sherlock G. 2000. Gene ontology: tool for the unification of biology. Nat Genet 25:25–29. doi:10.1038/75556. 10802651 PMC3037419

[42] The Gene Ontology Consortium. 2019. The Gene Ontology Resource: 20 years and still GOing strong. Nucleic Acids Res 47:D330–D338. doi:10.1093/nar/gky1055. 30395331 PMC6323945

[43] Perez-Riverol Y, Csordas A, Bai J, Bernal-Llinares M, Hewapathirana S, Kundu DJ, Inuganti A, Griss J, Mayer G, Eisenacher M, Perez E, Uszkoreit J, Pfeuffer J, Sachsenberg T, Yilmaz S, Tiwary S, Cox J, Audain E, Walzer M, Jarnuczak AF, Ternent T, Brazma A, Vizcaino JA. 2019. The PRIDE database and related tools and resources in 2019: improving support for quantification data. Nucleic Acids Res 47:D442–D450. doi:10.1093/nar/gky1106. 30395289 PMC6323896

