# Supplementary material for: Biosafety and Proteome Profiles of Different Heat Inactivation Methods for Mycobacterium tuberculosis
Source: Microbiol Spectr. 2021 Dec 22;9(3):e00716-21. doi: 10.1128/spectrum.00716-21 (PMC8694153; doi:10.1128/spectrum.00716-21)
Supplement: SUPPLEMENTAL FILE 1 — Supplemental material. Download SPECTRUM00716-21_Supp_1_seq9.pdf, PDF file, 0.2 MB [file spectrum00716-21_supp_1_seq9.pdf]

Supplementary Table 1. List of proteins uniquely identified in 90-mins of heat-inactivation

Protein IDs	Protein name	Peptides	Razor + unique peptides	Unique peptides	Sequence coverage [%]	Score	Mean LFQ intensity 90 mins
tr|I6WYY7|I6WYY7_MYCTU	(3R)-hydroxyacyl-ACP dehydratase subunit HadB	4	4	4	51	15	1377850
sp|P9WH65|RS11_MYCTU	30S ribosomal protein S11	2	2	2	12	12	3729150
sp|P9WH55|RS15_MYCTU	30S ribosomal protein S15	4	4	4	48	28	3206600
sp|P9WH39|RS2_MYCTU	30S ribosomal protein S2	2	2	2	15	103	14763500
sp|P9WH35|RS4_MYCTU	30S ribosomal protein S4	6	6	6	34	62	3650167
sp|P9WH33|RS5_MYCTU	30S ribosomal protein S5	7	7	7	38	28	2446850
sp|P9WH25|RS9_MYCTU	30S ribosomal protein S9	3	3	3	19	8	1577450
sp|P9WNP7|FADB2_MYCTU	3-hydroxybutyryl-CoA dehydrogenase	3	3	3	14	13	365320
sp|P9WQD7|KASB_MYCTU	3-oxoacyl-[acyl-carrier-protein] synthase 2	5	5	5	25	38	3047967
sp|P9WHE1|RL13_MYCTU	50S ribosomal protein L13	3	3	3	22	36	2096950
sp|P9WHD1|RL18_MYCTU	50S ribosomal protein L18	2	2	2	28	13	1152600
sp|P9WHB9|RL23_MYCTU	50S ribosomal protein L23	2	2	2	37	12	502505
sp|P9WHB3|RL27_MYCTU	50S ribosomal protein L27	3	3	3	26	18	2124900
sp|P9WH81|RL6_MYCTU	50S ribosomal protein L6	2	2	2	17	8	1614150
sp|O53166|ACNA_MYCTU	Aconitate hydratase A	12	12	12	18	72	3195767
sp|P9WID5|ADOK_MYCTU	Adenosine kinase	3	3	3	20	11	328265
tr|L7N655|L7N655_MYCTU	Alanine aminopeptidase	2	2	2	3	68	1663250
sp|P9WMK1|ACR_MYCTU	Alpha-crystallin	4	4	4	52	34	3340150
sp|P9WN51|GCST_MYCTU	Aminomethyltransferase	3	3	3	14	28	739685
sp|P9WPU9|ATPG_MYCTU	ATP synthase gamma chain	5	5	5	23	56	706690
sp|P9WPC9|CLPC1_MYCTU	ATP-dependent Clp protease ATP-binding subunit ClpC1	3	3	3	6	22	1099500
sp|O05779|FTSE_MYCTU	Cell division ATP-binding protein FtsE	4	4	4	22	18	592095
sp|P9WJF3|CWSA_MYCTU	Cell wall synthesis protein CwsA	3	3	3	39	15	1682700
sp|P9WNV9|DNAJ1_MYCTU	Chaperone protein DnaJ 1	4	4	4	17	24	525827
sp|P9WMJ7|HTPG_MYCTU	Chaperone protein HtpG	3	3	3	5	22	701355
tr|I6XF60|I6XF60_MYCTU	Conserved alanine and arginine rich protein	2	2	2	7	13	1129900
tr|I6YF16|I6YF16_MYCTU	Conserved protein	4	4	4	56	37	1741300
tr|I6YGW9|I6YGW9_MYCTU	Conserved protein	5	5	5	52	27	622173
tr|O06216|O06216_MYCTU	Conserved protein	4	4	4	21	30	1315100
tr|O06310|O06310_MYCTU	Conserved protein	3	3	3	21	29	751820
tr|O53777|O53777_MYCTU	Conserved protein	2	2	2	17	26	592085
tr|O53979|O53979_MYCTU	Conserved protein	2	2	2	11	57	530465
tr|O86353|O86353_MYCTU	Conserved protein	2	2	2	13	23	447765
tr|P95198|P95198_MYCTU	Conserved protein	2	2	2	15	10	567780

tr|P95270|P95270_MYCTU	Conserved protein	2	2	2	20	24	228495
sp|P9WP69|COX2_MYCTU	Cytochrome c oxidase subunit 2	1	1	1	5	7	3613000
sp|P9WQP3|A85A_MYCTU	Diacylglycerol acyltransferase/mycolyltransferase Ag85A	2	2	2	7	12	7313300
sp|P9WHH9|DLDH_MYCTU	Dihydrolipoyl dehydrogenase	4	4	4	16	24	398800
tr|I6XW38|I6XW38_MYCTU	DNA topoisomerase (ATP-hydrolyzing)	2	2	2	11	42	140117
sp|P9WG49|TOP1_MYCTU	DNA topoisomerase 1	4	4	4	7	32	419720
sp|O53509|Y2175_MYCTU	DNA-binding protein Rv2175c	3	3	3	30	25	602525
sp|P9WGZ1|RPOA_MYCTU	DNA-directed RNA polymerase subunit alpha	5	5	5	20	48	2041550
sp|P9WNM7|EFG_MYCTU	Elongation factor G	6	6	6	15	20	1029045
sp|P9WNM1|EFTS_MYCTU	Elongation factor Ts	4	4	4	23	21	1231750
sp|P9WNR7|ECCB1_MYCTU	ESX-1 secretion system ATPase EccB1	3	3	3	9	43	1099550
sp|P9WNR3|ECCB3_MYCTU	ESX-3 secretion system ATPase EccB3	7	7	7	21	33	1702380
sp|P9WJE5|ECCE3_MYCTU	ESX-3 secretion system protein EccE3	2	2	2	8	28	1519450
sp|P9WNE5|BFRB_MYCTU	Ferritin BfrB	4	4	4	31	37	5407367
sp|P9WQA3|ALF_MYCTU	Fructose-bisphosphate aldolase	3	3	3	13	24	1773167
sp|P9WMR9|DHAA_MYCTU	Haloalkane dehalogenase 3	2	2	2	9	12	503060
tr|O53673|O53673_MYCTU	Heat shock protein Hsp (Heat-stress-induced ribosome-binding protein A)	2	2	2	23	20	2986050
tr|O53611|O53611_MYCTU	Isocitrate dehydrogenase [NADP]	4	4	4	11	41	1559667
sp|P9WHR7|LEXA_MYCTU	LexA repressor	2	2	2	10	14	830455
sp|P9WIT3|GULDH_MYCTU	L-gulono-1,4-lactone dehydrogenase	6	6	6	19	29	2354450
sp|P9WK55|LPRA_MYCTU	Lipoprotein LprA	4	4	4	21	43	6101867
tr|Q79FZ9|Q79FZ9_MYCTU	Mce-family protein Mce1A	4	4	4	13	20	1457550
tr|O07415|O07415_MYCTU	Mce-family protein Mce1C	3	3	3	10	93	958380
tr|O07416|O07416_MYCTU	Mce-family protein Mce1D	2	2	2	5	19	1846550
tr|L0T2W6|L0T2W6_MYCTU	Mce-family protein Mce1F	10	10	10	26	49	4600900
sp|P9WIV5|NUOE_MYCTU	NADH-quinone oxidoreductase subunit E	2	2	2	19	6	1178850
sp|P9WGU1|PSTS1_MYCTU	Phosphate-binding protein PstS 1	4	4	4	22	50	843697
sp|O53512|AROG_MYCTU	Phospho-2-dehydro-3-deoxyheptonate aldolase AroG	2	2	2	6	15	963420
sp|P9WID1|PGK_MYCTU	Phosphoglycerate kinase	6	6	6	21	41	1928650
sp|P9WQ73|SERC_MYCTU	Phosphoserine aminotransferase	3	3	3	15	18	541920
tr|O33272|O33272_MYCTU	Possible conserved membrane or secreted protein	4	4	4	29	11	1538100
tr|I6XF52|I6XF52_MYCTU	Possible conserved transmembrane alanine and glycine rich protein	4	4	4	12	15	790613
tr|O06624|O06624_MYCTU	Possible Inv protein	4	4	4	36	49	763947
tr|Q10782|Q10782_MYCTU	Possible ketoacyl reductase	2	2	2	6	4	1266600
tr|L0T7Y7|L0T7Y7_MYCTU	PPE family protein PPE31 (Fragment)	1	1	1	5	7	2418250
tr|I6Y7V6|I6Y7V6_MYCTU	Probable acyl-CoA ligase FadD31 (Acyl-CoA synthetase) (Acyl-CoA synthase)	5	5	5	13	85	1002847
tr|O53422|O53422_MYCTU	Probable beta-ketoacyl CoA thiolase FadA3	7	7	7	27	31	1784100

tr|P96886|P96886_MYCTU	Probable bifunctional protein acetyl-/propionyl-coenzyme A carboxylase (Epsilon chain) AccE5	1	1	1	12	15	2974533
tr|I6X7X3|I6X7X3_MYCTU	Probable conserved lipoprotein LpqG	3	3	3	23	21	3451033
tr|O07424|O07424_MYCTU	Probable conserved transmembrane protein	2	2	2	6	11	372980
tr|I6Y4E8|I6Y4E8_MYCTU	Probable enoyl-CoA hydratase EchA5 (Enoyl hydrase) (Unsaturated acyl-CoA hydratase) (Crotonase)	3	3	3	16	22	890020
sp|P9WNN9|ECHA8_MYCTU	Probable enoyl-CoA hydratase echA8	6	6	6	24	45	2647450
sp|O69687|FAMT_MYCTU	Probable fatty acid methyltransferase Rv3720	3	3	3	10	28	932250
tr|P95029|P95029_MYCTU	Probable fatty acid synthase Fas (Fatty acid synthetase)	4	4	4	3	14	712520
tr|I6XFB7|I6XFB7_MYCTU	Probable membrane protein	3	3	3	21	24	1372100
tr|I6Y231|I6Y231_MYCTU	Probable multifunctional mycocerosic acid synthase membrane-associated Mas	3	3	3	3	22	1448467
sp|P9WHW1|PPIB_MYCTU	Probable peptidyl-prolyl cis-trans isomerase B	3	3	3	19	22	863395
tr|O50460|O50460_MYCTU	Probable short-chain type dehydrogenase/reductase	5	5	5	23	48	1884700
tr|O53669|O53669_MYCTU	Probable succinate dehydrogenase [iron-sulfur subunit] (Succinic dehydrogenase)	2	2	2	12	6	891595
tr|Q79FN7|Q79FN7_MYCTU	Probable transcriptional regulatory protein MoxR1	7	7	7	28	30	1636567
sp|P9WGA5|Y2603_MYCTU	Probable transcriptional regulatory protein Rv2603c	4	4	4	28	23	1569250
sp|O53554|ILVX_MYCTU	Putative acetolactate synthase large subunit IlvX	4	4	4	12	51	1093975
sp|P9WNZ7|DESA1_MYCTU	Putative acyl-[acyl-carrier-protein] desaturase DesA1	2	2	2	9	4	423840
sp|P9WNZ5|DESA2_MYCTU	Putative acyl-[acyl-carrier-protein] desaturase DesA2	4	4	4	27	18	912735
sp|I6WXS6|VPB51_MYCTU	Putative antitoxin VapB51	2	2	2	59	20	1671550
sp|P9WJZ7|Y1220_MYCTU	Putative O-methyltransferase Rv1220c	3	3	3	23	8	671730
sp|P9WK65|LPPX_MYCTU	Putative phthiocerol dimycocerosate transporter LppX	4	4	4	23	40	2035300
sp|P9WNX7|GABD2_MYCTU	Putative succinate-semialdehyde dehydrogenase [NADP(+)] 2	3	3	3	10	8	1026560
sp|P9WHF9|THTR_MYCTU	Putative thiosulfate sulfurtransferase	3	3	3	16	8	2265600
sp|P9WHF7|THT2_MYCTU	Putative thiosulfate sulfurtransferase SseA	3	3	3	15	17	1674200
sp|P9WIS9|ODP1_MYCTU	Pyruvate dehydrogenase E1 component	3	3	3	4	13	2955300
sp|P9WGV1|METK_MYCTU	S-adenosylmethionine synthase	5	5	5	27	20	414847
sp|P9WI79|PKND_MYCTU	Serine/threonine-protein kinase PknD	5	5	5	17	31	1217550
sp|P9WGC7|SUCD_MYCTU	Succinate--CoA ligase [ADP-forming] subunit alpha	4	4	4	25	8	2320850
sp|P9WG25|TKT_MYCTU	Transketolase	5	5	5	11	29	1788100
sp|P9WKK1|IF2_MYCTU	Translation initiation factor IF-2	7	7	7	9	45	4497133
sp|P9WKJ9|IF3_MYCTU	Translation initiation factor IF-3	6	6	6	42	22	1371750
sp|P9WG43|TPIS_MYCTU	Triosephosphate isomerase	3	3	3	22	7	954585
sp|P9WG15|Y2563_MYCTU	Uncharacterized ABC transporter permease Rv2563	3	3	3	12	43	2756150
sp|P9WQA5|Y2971_MYCTU	Uncharacterized oxidoreductase Rv2971	5	5	5	23	31	1462000
tr|I6X824|I6X824_MYCTU	Uncharacterized protein	1	1	1	36	17	455835
tr|O05592|O05592_MYCTU	Uncharacterized protein	4	4	4	12	17	1292400

sp|P9WM73|Y088_MYCTU	Uncharacterized protein Rv0088	4	4	4	20	18	6896433
sp|P9WKS5|Y634A_MYCTU	Uncharacterized protein Rv0634A	3	3	3	59	15	8962400
sp|P9WKP5|Y898_MYCTU	Uncharacterized protein Rv0898c	2	2	2	33	71	2900100
sp|P9WLA9|Y2411_MYCTU	Uncharacterized protein Rv2411c	3	3	3	9	8	745500
sp|P9WL61|Y2632_MYCTU	Uncharacterized protein Rv2632c	2	2	2	30	21	1118015
sp|P9WKW7|Y3788_MYCTU	Uncharacterized protein Rv3788	4	4	4	39	11	1604050
sp|P9WFN1|Y2140_MYCTU	UPF0098 protein Rv2140c	3	3	3	18	19	3746150
sp|P9WFL3|Y3193_MYCTU	UPF0182 protein Rv3193c	6	6	6	11	25	639030
